# Branch retinal artery occlusion in a young woman with papilledema and a prepapillary vascular loop

**DOI:** 10.1016/j.ajoc.2025.102286

**Published:** 2025-02-19

**Authors:** Damian Jaggi, Hilary M. Grabe

**Affiliations:** a Department of Ophthalmology, Inselspital, Bern University Hospital, University of Bern, Bern, Switzerland; b Department of BioMedical Research, University of Bern, Bern, Switzerland

**Keywords:** Prepapillary vascular loops, Papilledema, Branch retinal artery occlusion, Fluorescein angiography

## Abstract

**Purpose:**

To report a case of a branch retinal artery occlusion (BRAO) associated with a prepapillary vascular loop in a patient with papilledema.

**Observations:**

A 22-year-old woman presented with sudden superior visual field loss in the left eye. Initial examination demonstrated mild optic disc edema bilaterally. The diagnosis of idiopathic intracranial hypertension was made and treatment with acetazolamide was initiated. During follow-up examination bilateral prepapillary vascular arterial loops and a BRAO in the left eye were diagnosed. Over time the initially dense superior arcuate visual field defect improved.

**Conclusions and importance:**

We report a case of BRAO and prepapillary loops in a patient with coexisting papilledema. The initial symptoms of unilateral visual field loss were falsely attributed to the presence of papilledema and the BRAO was only detected on subsequent examination. The case highlights the importance of correlating the presenting chief complaint with the examination findings to reach the correct diagnosis.

## 1 Introduction

Prepapillary vascular loops are rare anomalies of the retinal vessels, which may be unilateral or bilateral. There is significant variation in the location and shape of these vascular loops producing various branching patterns and affecting the vessel course, tortuosity, with aberrant macular or ciliary arteries, crossing, pseudoaneurysms, or prepapillary loops.^1^, ^2^, ^3^, ^4^, ^5^ In spite of the mostly asymptomatic and benign presentation, complications such as branch or central retinal artery occlusion and vitreous hemorrhage have been described.^1^^,^^6^^,^^7^ The median age of onset of these complications is usually lower than in artery occlusions in patients with a higher cardiovascular risk. Since the artery occlusion in these cases is associated with an anatomical and not with cardiovascular risk factors, there is no established treatment paradigm.^3^

## 2 Case report

A 22-year-old female patient with no significant past medical history presented for urgent evaluation with a sudden and painless loss of her superior visual field of the left eye, which she noticed approximately 8 hours prior to presentation. She denied a history of trauma. She reported a severe headache with nausea the previous night, which resolved after self-induced vomiting: there was no temporal correlation between the visual field loss and vomiting episode. She weighed 141 pounds with a height of 5.15 feet (BMI 26). On initial examination, her visual acuity was 20/20 in both eyes. Pupil examination revealed a left RAPD. Intraocular pressure and anterior segment examination were unremarkable. Fundus examination revealed mild optic disc edema in both eyes with a peripapillary flame shaped hemorrhage in the left eye. Based on the bilateral optic disc edema, idiopathic intracranial hypertension was suspected and the patient was referred for urgent MRI and neurology evaluation with lumbar puncture.

MRI demonstrated bilateral dilation of the optic nerve sheath and excluded other intracranial pathology. A lumbar puncture was performed with an opening pressure of 41 mm Hg (normal range 4.4–18.4 mm Hg/6–25 cm H_2_O^8^) and normal CSF composition confirming the diagnosis of idiopathic intracranial hypertension. The patient was started on acetazolamide 1000 mg per day.

**Fig. 1:**
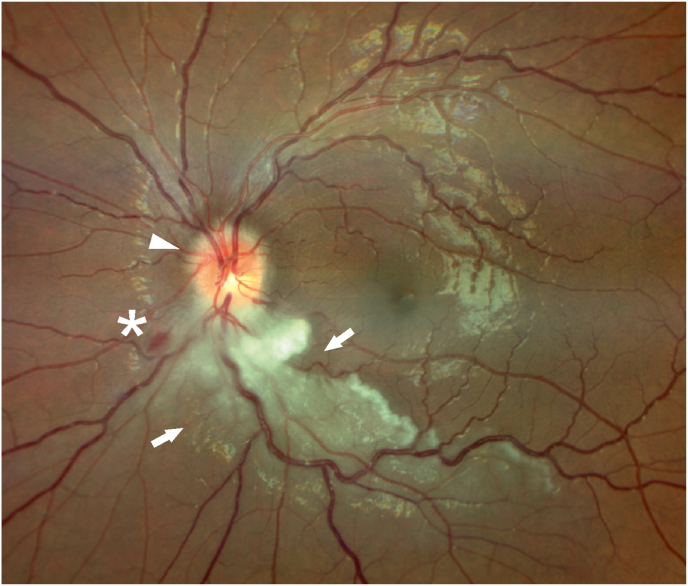
Color fundus photograph of the left eye, showing a BRAO of the inferior vascular arcade, with ischemic edematous retina (arrows) and a small retinal hemorrhage (asterisk). Mild optic disc edema can be seen (arrowhead). (For interpretation of the references to colour in this figure legend, the reader is referred to the Web version of this article.)

At the follow-up examination 4 days later the patient reported no significant change in her visual field loss. Visual acuity remained 20/20 in both eyes. Formal visual field testing (Octopus) demonstrated a dense superior arcuate defect extending nearly to the horizontal midline. Fundus examination revealed a prepapillary vascular loop in both eyes with an edematous retina along the inferotemporal arcade in the left eye, in addition to the preexisting mild papilledema (Fig. 1).

**Fig. 2:**
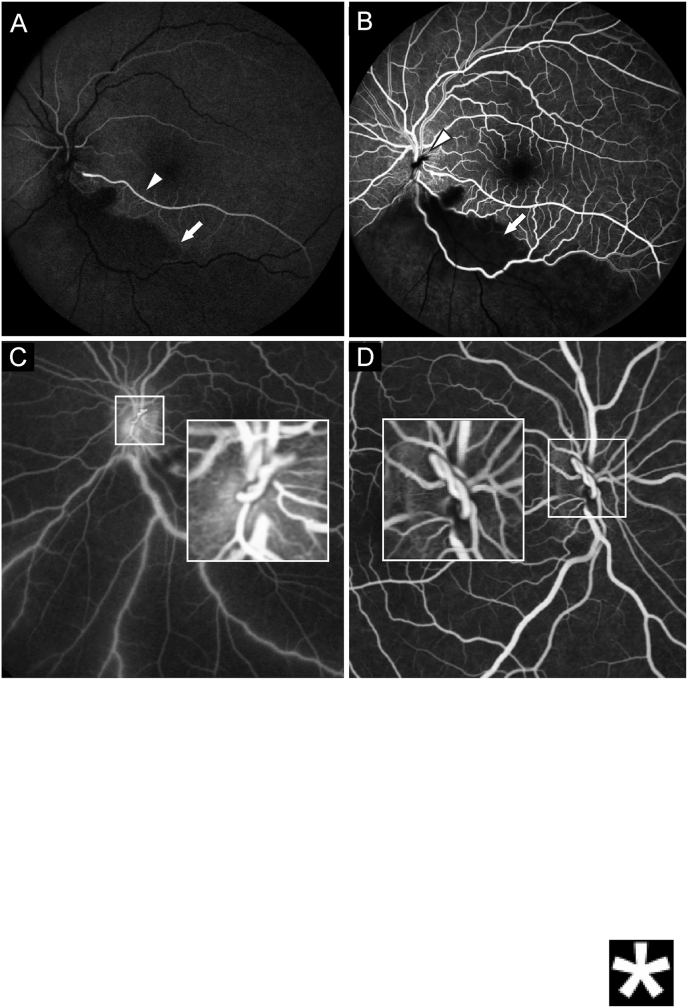
Fluorescein-angiography of the left eye (A–C). A shows the presence of a cilioretinal artery (arrowhead) and blockage of the background fluorescence (arrow) in the infarcted area. B confirms the non-perfusion of the inferior retina (arrow) and non-perfusion of the vascular loop (arrowhead). C confirms the vascular loop and retrograde filling of the retinal arteries and loop. D shows the late venous phase of the fluorescein-angiography of the right eye. The prepapillary vascular loop is present in a similar configuration, but retinal perfusion is normal.

We diagnosed an inferior branch retinal artery occlusion (BRAO) of the left eye, corresponding to the visual field defect. In addition, we performed Fluorescein-angiography (Fig. 2), which revealed a cilioretinal artery and some residual perfusion of the infarcted area. The prepapillary vascular loop was confirmed as the site of the arterial obstruction.

**Fig. 3:**
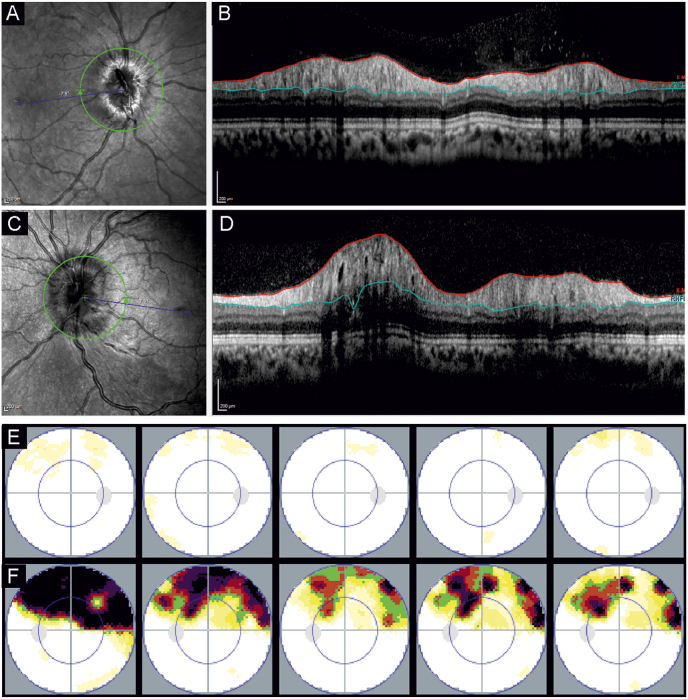
A shows the infrared image of the optic disc of the right eye, and B shows the peripapillary swollen retinal nerve fiber layer (RNFL). C and D show the left eye correspondingly, with more pronounced swelling in the RNFL. E and F show the static visual field measurements over time (months 0, 1, 2, 4,.16) of the right and left eye, respectively. The initial dense superior arcuate defect of the left eye recovers significantly over time.

Over time the visual field defect improved, although a residual superior arcuate defect remained (Fig. 3). This visual field defect remain symptomatic for the patient. We continued treatment with acetazolamide until the papilledema resolved. Mild papilledema recurred following cessation of treatment, which was initially treated with acetazolamide. Due to treatment side effects, we discontinued acetazolamide and encouraged weight loss with continued follow-up examinations.

## 3 Discussion

We report a case of a branch retinal artery occlusion in a patient with prepapillary vascular loops and papilledema. Due to the presence of bilateral optic disc edema, the branch retinal artery occlusion was initially overlooked and her symptoms were falsely attributed to idiopathic intracranial hypertension, despite her reported visual field loss not being consistent with mild papilledema. This case illustrates an important teaching point that the examination findings must be considered in the context of the patient's history and symptoms to reach the correct diagnosis.

In this case, the visual field loss is clearly a result of the branch retinal occlusion and not papilledema. Mild papilledema, such as seen in this case (Fig. 3) is often an incidental finding with no or minimal visual symptoms. When present, visual symptoms may include blurred vision, photopsias, transient visual obscurations, and diplopia. Systemic symptoms of headache, tinnitus, and interscapular pain can be vague or absent.^9^

Interestingly, the significance of the much rarer finding of a prepapillary loop in both eyes and the associated risk for a BRAO was initially overlooked. Complications from these loops, such as retinal artery occlusions typically occur spontaneously. The younger age and absent cardiovascular risk profile in these patients is suggestive for mechanical complications of these loops, rather than thromboembolic events. Supporting the theory of a mechanical cause, traumatic torsion of the vasculature with consecutive infarction has been described in one case.^7^

In our patient, it is unclear if the papilledema increased the risk of an arterial occlusion associated with the prepapillary vascular loop. Theoretically, papilledema should be more likely to lead to venous, rather than arterial, obstruction given the potential compressive effect on the retinal veins as they cross the optic nerve. In our case, the mild papilledema is most likely an incidental finding unrelated to the branch retinal occlusion.

## 4 Conclusion

This case describes a unique combination of a rare prepapillary vascular loop as a risk factor for BRAO in a young patient with coincidental mild papilledema. This case highlights the importance of the patient history when assessing examination findings, particularly in the urgent clinical setting where incidental findings must be quickly differentiated from other pathologic findings responsible for patients’ symptoms.

## CRediT authorship contribution statement

**Damian Jaggi:** Writing – original draft, Investigation, Data curation, Conceptualization. **Hilary M. Grabe:** Writing – review & editing, Supervision, Project administration, Investigation, Conceptualization.

## Patient consent

A written consent for the use of data was obtained.

## Financial support

None.

## Declaration of competing interest

The authors declare that they have no known competing financial interests or personal relationships that could have appeared to influence the work reported in this paper.
